# Salivary ^1^H NMR metabolic profiling of diabetic patients with and without periodontitis: an exploratory pilot study

**DOI:** 10.1186/s44342-026-00085-4

**Published:** 2026-10-26

**Authors:** Samaneh Vaziri-Amjad, Bahar Zalei, Nazli Rabienejad, Ali Golshaian, Amir Taherkhani

**Affiliations:** 1 Dental Implants Research Center, Avicenna Institute of Clinical Sciences, Hamadan University of Medical Sciences, Hamadan, Iran; 2 Student Research Committee, Hamadan University of Medical Sciences, Hamadan, Iran; 3 Department of Endocrinology, School of Medicine, Hamadan University of Medical Sciences, Hamadan, Iran; 4 Research Center for Molecular Medicine, Institute of Cancer, Hamadan University of Medical Sciences, Hamadan, Iran; 5 Urology and Nephrology Research Center, Avicenna Institute of Clinical Sciences, Hamadan University of Medical Sciences, Hamadan, Iran

**Keywords:** Biomarkers, Diabetes mellitus, Metabolomics, Nuclear magnetic resonance, Periodontitis, Saliva

## Abstract

**Background:**

Periodontitis is a widespread chronic inflammatory condition that frequently coexists with diabetes mellitus (DM). Identifying non-invasive biomarkers is essential for early detection, targeted therapy, and better understanding of the bidirectional relationship between these two diseases. This study aimed to investigate salivary metabolomic profiles in diabetic individuals with and without periodontitis to identify candidate metabolites associated with periodontitis in patients with diabetes.

**Methods:**

This pilot study compared salivary ^1^H NMR metabolomic profiles of patients with diabetes with and without periodontitis (*n* = 13 per group). Unstimulated whole saliva was collected from both groups. Partial least squares discriminant analysis (PLS-DA) was applied to differentiate between the groups. Spectral variables with VIP > 1 were selected for metabolite annotation, followed by metabolite set enrichment analysis (MSEA) to identify dysregulated metabolic pathways.

**Results:**

Using the PLS-DA model, a total of 78 discriminating variables were identified, leading to the annotation of ten key metabolites: glycolic acid, thymidine, tyramine, deoxyguanosine, cytidine, uridine, NADPH, ADP, serotonin, and nicotinate. MSEA revealed significant pathway disturbances, with pyrimidine metabolism emerging as a notably enriched pathway.

**Conclusion:**

The metabolomics study of saliva using ^1^H NMR showed distinct metabolic disturbances, particularly in pyrimidine metabolism, in diabetic periodontitis. Although these altered metabolites could serve as potential candidates for non-invasive biomarkers, independent validation from a large cohort is still required before they can be translated into clinical diagnostic use.

**Supplementary Information:**

The online version contains supplementary material available at https://doi.org/10.1186/s44342-026-00085-4.

## Introduction

Periodontitis is a chronic, progressive inflammatory condition that targets the tooth-supporting structures. It is characterized by gingival inflammation, periodontal pocket formation, and progressive destruction of periodontal soft tissues and alveolar bone, ultimately leading to tooth mobility and loss [1, 2]. This disease is a major global public health challenge; recent estimates indicate that periodontitis affects about 62% of dentate adults worldwide, with severe cases accounting for 23.6% of this population [3, 4]. The disease’s pathogenesis is highly multifactorial, driven by complex interactions among microbial dysbiosis, host immune responses, genetic predispositions, and environmental or behavioral factors such as smoking and poor oral hygiene [5, 6]. Given its widespread prevalence and destructive nature, preventing, early diagnosing, and targeting periodontitis are critical public health priorities [7, 8].

Beyond its localized impact on the oral cavity, periodontitis is increasingly recognized as a condition with profound systemic consequences. It is intimately linked to a variety of systemic conditions, including cardiovascular diseases, obesity, Alzheimer’s disease, and adverse pregnancy outcomes [9]. In fact, periodontitis is now considered an independent risk factor for cardiovascular disease [10] and has been shown to influence the tumor microenvironment, potentially impacting cancer development [11]. Because periodontitis plays a central role in the interplay between oral and systemic health, treating the condition is vital to preserving patients’ overall well-being and underscores the need for individualized therapeutic approaches [4, 12].

Among these systemic connections, the association between periodontitis and diabetes mellitus (DM) is particularly critical. The two conditions share a well-documented, bidirectional relationship in which each exacerbates the other’s progression. Poorly controlled diabetes, in particular, significantly elevates the risk, severity, and rapid progression of periodontal tissue destruction through a multitude of complex biological mechanisms [13]. Despite this known clinical association, the precise molecular alterations underlying the exacerbation of periodontitis in diabetic patients remain incompletely understood [14].

Saliva is a highly informative, non-invasive biofluid that reflects both local oral pathology and systemic metabolic shifts [15, 16]. Nuclear magnetic resonance (NMR) spectroscopy is a powerful analytical tool that elucidates molecular architecture and dynamic behavior at atomic resolution while enabling quantitative profiling of complex biological samples. Because it is non-invasive and requires minimal sample preparation, it is particularly well-suited for applications in biomedicine and metabolomics [17–19]. Therefore, we used NMR spectroscopy to analyze the salivary metabolome of patients with DM and periodontitis and compared it with that of DM patients without periodontitis. By identifying differentially expressed metabolites (DEMs) and conducting pathway enrichment analysis, this study aims to elucidate the underlying metabolic pathways driving periodontitis in the context of DM.

## Materials and methods

### Participants and eligibility criteria

Participants: Adults diagnosed with diabetes mellitus were recruited from the School of Dentistry at Hamadan University of Medical Sciences, Hamadan, Iran, between February 2025 and April 2026. Enrolled individuals were divided into two groups: those with diabetes and periodontitis (case group) and those with diabetes but no periodontitis (control group). This pilot study included 13 participants per group.

Inclusion criteria: age ≥18 years; confirmed diagnosis of diabetes mellitus (type 1 or 2).

Exclusion criteria: pregnancy; use of antibiotics or anti-inflammatory agents within the 3 months before enrollment; presence of other major systemic diseases.

Diabetes mellitus diagnosis: Participants had a documented diagnosis of diabetes mellitus (type 1 or 2), confirmed by HbA1c ≥6.5% and fasting plasma glucose ≥126 mg/dL.

Periodontitis diagnosis: Periodontitis was diagnosed according to the 2017 World Workshop on the Classification of Periodontal and Peri-Implant Diseases and Conditions [20]. Participants assigned to the periodontitis group met at least one of the following criteria:

- Interdental clinical attachment loss (CAL) detectable at ≥2 non-adjacent teeth, or
- Buccal or oral CAL ≥3 mm with pocket depth >3 mm at ≥2 teeth.

Radiographic bone loss was also assessed where available. Control participants exhibited no clinical or radiographic evidence of periodontitis.

Demographic and clinical data: Information on age, gender, smoking status, type of diabetes, disease duration, HbA1c levels, and current medications was collected using a standardized questionnaire and verified through medical records.

### Sample collection and preprocessing

A cross-sectional observational study comparing two groups of diabetic patients was conducted. All participants provided informed consent before inclusion. Standardized questionnaires were then used to record participants’ clinical and demographic profiles. Unstimulated whole saliva samples were collected from volunteers at Hamadan University of Medical Sciences (Hamadan, Iran) between February 2025 and April 2026. The ^1^H NMR study cohorts included 13 diabetic patients with periodontitis (case group) and 13 diabetic patients without periodontitis (control group).

To mitigate the influence of circadian rhythms, all collections were conducted in the morning between 9:00 and 11:00 [21–23]. Participants were required to abstain from food, beverages, caffeine, alcohol, nicotine, chewing gum, and all oral hygiene practices for at least 1 h before sampling [22, 24].

The collection protocol involved an initial mouth rinse with deionized water followed by a 10-min rest period. Participants then provided saliva via passive drooling into ice-chilled polypropylene tubes. Saliva produced during the first minute was discarded, while the output from the subsequent 5 min was retained. The samples were transported on ice within 120 min of collection [25]. To inhibit microbial proliferation, sodium azide (NaN_3_) was added as a preservative to achieve a final concentration of 0.02% (w/v). Finally, the specimens were centrifuged at 10,000 × g for 10 min at 4 °C [26], and the resulting supernatants were aliquoted and frozen at −80 °C until further analysis.

To minimize potential bias, all saliva samples were obtained under uniform conditions, and spectral processing was carried out using the same parameters for every sample. The clinician responsible for periodontal examinations was blinded to the NMR and metabolomics data, and the analyst performing spectral processing was unaware of group allocation.

### ^*1*^*H NMR analysis*

For ^1^H NMR analysis, 675 µL of saliva was combined with 90 µL of phosphate buffer (prepared in D_2_O, deuterium oxide, 99.9% D, Aldrich Chemicals Company) and 60 µL of D_2_O. The mixture was then transferred to a 5-mm NMR tube. ^1^H resonance spectra were acquired at 298 K (25 °C) using a German Bruker 400-MHz NMR spectrometer. To suppress signals from macromolecules, the Carr–Purcell–Meiboom–Gill (CPMG) pulse sequence was employed. Spectral processing—including binning, normalization, and chemical shift alignment—was performed using ProMetab software (version Prometab_v3_3) [27] within the MATLAB environment (version 9.1; MathWorks, Cambridge, UK). The spectral range from 0.2 to 10 ppm was divided into buckets of 0.05 ppm width, with the water resonance excluded from the analysis. Quantile normalization was performed on the data using the ProMetab software.

### Statistical and chemometric analysis

The normality of the HbA1c values in each group was determined using the Shapiro–Wilk test. Since the distribution was normal in both groups (*p* > 0.05), an independent-samples *t*-test (with Welch’s correction, as available in R version 4.4.1) was used to compare the HbA1c levels between the groups. A *p* value < 0.05 was considered statistically significant.

Partial least squares discriminant analysis (PLS-DA), an advanced supervised multivariate modeling technique, was conducted using XLSTAT software (Addinsoft, New York, NY, USA) using a single-component architecture. Metabolite variables demonstrating a variable importance in the projection (VIP) score of >1 were deemed statistically significant. To assess the PLS-DA model’s diagnostic performance and predictive capability, a receiver operating characteristic (ROC) curve was subsequently generated. Also, to thoroughly evaluate the robustness of the model and to counter the risk of overfitting that is typical in small-cohort metabolomics studies, an internal cross-validation procedure based on a 7-fold cross-validation method was carried out. The predictive power of the model was calculated using the cross-validated predictive parameter (*Q*^2^).

### Permutation test

Model validity was assessed using a label-permutation test with the ropls R package. The test involved 500 permutations. The model used one component and 7-fold cross-validation. The original PLS-DA model was refit each time. Empirical *p* values (*pR*^2^*Y* and *pQ*^2^) were calculated by comparing the original metrics with those from the permuted distribution.

### Metabolite identification

Following the selection of spectral variables with a VIP score >1, a multi-step procedure was utilized for metabolite annotation. Initially, the chemical shift (ppm) of each significant variable was queried against the Biological Magnetic Resonance Data Bank (BMRB) [28] (https://bmrb.io/bmrb/citation.shtml) to compile a preliminary list of candidate metabolites. Next, Chenomx software (version 12.02) was employed to cross-reference the spectral signatures of these candidates against a representative sample selected as a reference spectrum.

Because individual metabolites typically produce multiple peaks across various chemical shifts, candidate identification was validated by assessing the overlap of all corresponding peaks with the experimental reference data. The candidate exhibiting the highest degree of peak alignment was consequently designated as the differentially expressed metabolite distinguishing diabetic patients with periodontitis from diabetic controls. It should be noted that the results section highlights the most distinct peak for each annotated metabolite, which may not exclusively correspond to the exact chemical shift initially flagged with a VIP > 1.

### Metabolite set enrichment analysis

To explore the biological pathways involved in periodontitis among individuals with diabetes, metabolite set enrichment analysis (MSEA) was carried out using the MetaboAnalyst 6.0 platform [29], accessible at https://www.metaboanalyst.ca/. The pathway enrichment results were derived from the final set of DEMs identified in diabetic patients with periodontitis relative to diabetic controls without periodontitis.

MSEA, conceptually analogous to gene set enrichment analysis (GSEA), is a tool tailored for interpreting metabolomic data. Rather than examining each metabolite in isolation and applying rigid cutoff thresholds, MSEA evaluates predefined collections of functionally linked metabolites as integrated units. This approach is particularly adept at capturing subtle but coordinated variations within metabolic pathways, changes that may be missed when using conventional, single-compound analytical strategies [30]. The false discovery rate (FDR) for pathway enrichment analysis was calculated using the Benjamini–Hochberg procedure to account for multiple testing across the identified metabolic pathways. The threshold for statistical significance was established at FDR < 0.05.

## Results

### Participants

A total of 30 diabetic patients were assessed for study eligibility. Of these, four were excluded: two did not meet the inclusion criteria because of hyperthyroidism, and two declined to participate. The remaining 26 participants were enrolled and allocated into two groups: 13 with periodontitis and 13 without periodontitis. All enrolled subjects provided saliva samples and were included in the final analysis. No exclusions were recorded after enrollment, and there was no loss to follow-up or missing data.

### Statistical matching of baseline characteristics

In both the periodontitis and control groups, HbA1c values followed a normal distribution (periodontitis group, Shapiro–Wilk *W* = 0.943, *p* = 0.495; control group, *W* = 0.954, *p* = 0.657). The two groups did not differ significantly in mean HbA1c (7.53% in the periodontitis group and 7.33% in the control group) (*t*(22.50) = 0.47, *p* = 0.642). The mean difference was 0.20 percentage points (95% CI −0.68 to 1.08). A summary of the participants’ characteristics is given in Table 1.

**Table 1:** Baseline demographic and clinical characteristics of participants with diabetes, with and without periodontitis (*n* = 13 per group)

Variable	Periodontitis (*n* = 13)	Control (*n* = 13)	*p*
Age (years)	56.2 ± 5.9	54.7 ± 8.6	0.6
Male sex, *n* (%)	8 (61.5)	8 (61.5)	1
HbA1c (%)	7.53 ± 1.21	7.33 ± 0.93	0.642

*HBA1c* hemoglobin A1c

### Variable importance in the projection

**Fig. 1:**
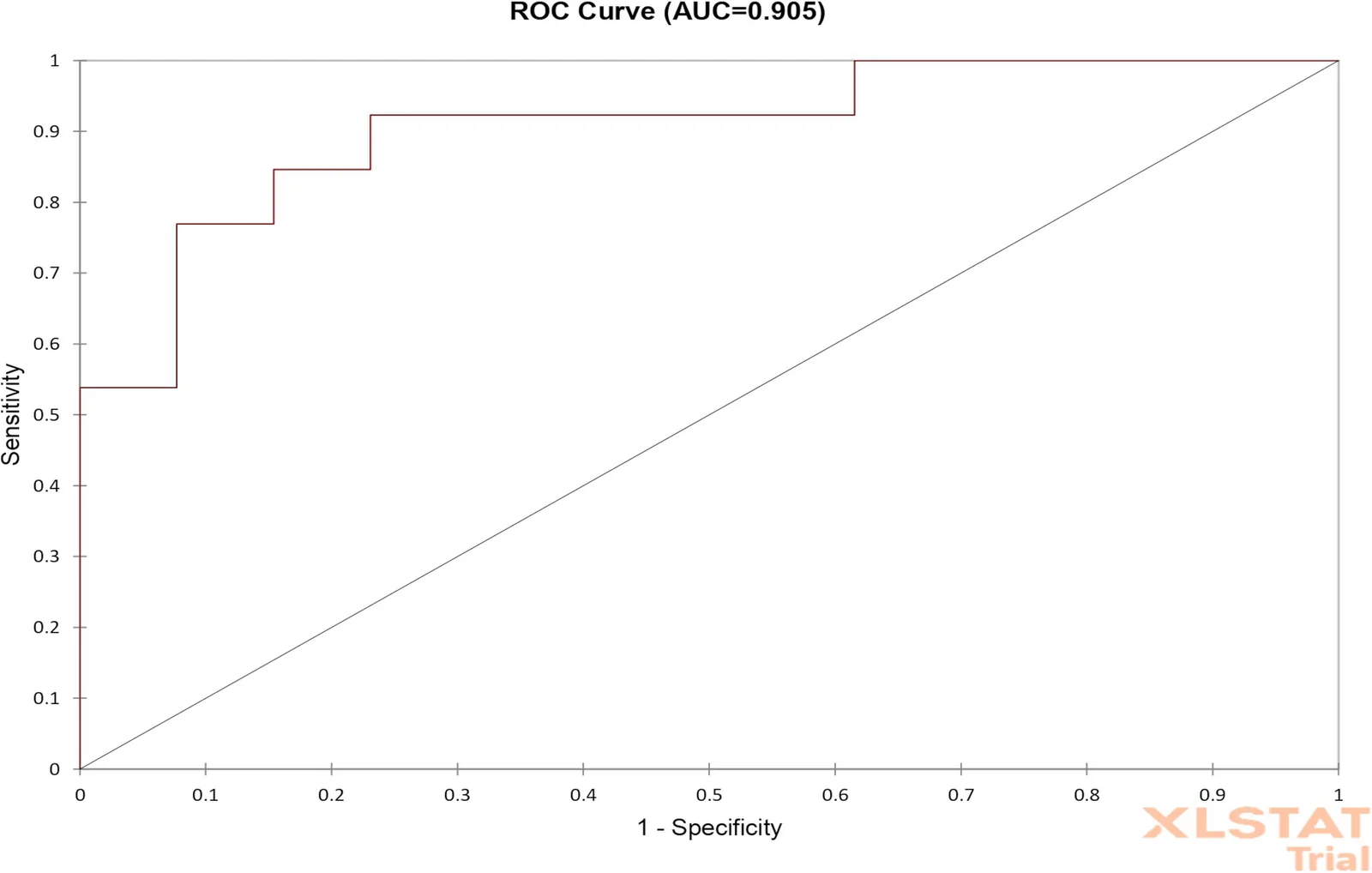
Receiver operating characteristic curve evaluating the diagnostic performance of the partial least squares discriminant analysis model. The area under the curve is 0.905

After preprocessing the ^1^H NMR raw data using the ProMetab software (Supplementary Data 1), a predictive model was constructed using PLS-DA applied to the ^1^H NMR spectral data. This model discriminated between diabetic patients with and without periodontitis. The PLS-DA model distinguished between the two groups (*R*^2^*X* = 0.449, *R*^2^*Y* = 0.146, *Q*^2^ = 0.031; Supplementary Data 2) and gave an apparent AUC of 0.905 (Fig. 1); however, its predictive ability was limited, and external validation is needed. Because the AUC was calculated from the training set, it is likely optimistic given the small sample size (13 per group) and the low *Q*^2^ value; therefore, it should be regarded as an apparent, not validated, estimate of performance. Using the PLS-DA diagnostic model, a total of 78 discriminating variables (chemical shifts in ppm) with VIP > 1 were identified when comparing the two cohorts and were retained for metabolite identification and biological pathway mapping. These differential variables, along with their corresponding effect sizes (Cohen’s *d*) and mean difference values (case mean − control mean), are presented in Table 2.

**Table 2:** PLS-DA analysis identified 78 variables (chemical shifts in ppm) with VIP values exceeding 1

ppm	VIP	(Mean case) − (mean control)	Effect size (Cohen’s *d*)
6.675	1.708	0.198	0.876
6.625	1.699	0.197	0.870
9.375	1.680	0.106	0.858
6.575	1.665	0.193	0.849
9.175	1.635	0.111	0.831
9.425	1.629	0.100	0.827
7.725	1.622	0.149	0.823
7.375	1.620	0.166	0.822
7.775	1.597	0.142	0.808
9.325	1.595	0.102	0.807
6.525	1.580	0.184	0.798
7.675	1.577	0.146	0.796
6.475	1.557	0.182	0.784
6.725	1.534	0.177	0.771
7.825	1.531	0.136	0.769
9.275	1.530	0.099	0.769
7.425	1.526	0.163	0.766
9.225	1.502	0.101	0.753
7.875	1.499	0.132	0.751
9.125	1.495	0.103	0.749
6.425	1.485	0.174	0.743
7.325	1.461	0.150	0.729
6.375	1.443	0.171	0.718
9.475	1.430	0.083	0.711
6.925	1.429	0.155	0.711
6.325	1.420	0.170	0.706
7.925	1.418	0.129	0.705
6.125	1.411	0.178	0.701
6.175	1.408	0.174	0.699
6.275	1.404	0.170	0.697
6.225	1.397	0.171	0.693
6.075	1.396	0.180	0.692
8.075	1.366	0.121	0.675
6.975	1.366	0.149	0.675
8.125	1.360	0.120	0.672
9.525	1.355	0.075	0.669
6.025	1.352	0.181	0.668
7.625	1.346	0.129	0.664
9.575	1.345	0.071	0.664
7.275	1.344	0.141	0.663
8.025	1.329	0.118	0.655
7.125	1.328	0.145	0.655
6.875	1.321	0.145	0.651
8.175	1.316	0.115	0.648
7.025	1.308	0.146	0.644
9.075	1.291	0.092	0.634
8.225	1.281	0.109	0.629
8.975	1.255	0.091	0.614
7.075	1.252	0.138	0.613
8.875	1.248	0.093	0.611
7.575	1.247	0.123	0.610
8.525	1.244	0.101	0.609
8.925	1.239	0.090	0.606
9.025	1.235	0.089	0.604
7.975	1.229	0.112	0.601
7.225	1.227	0.131	0.600
7.175	1.219	0.133	0.595
6.825	1.210	0.136	0.590
9.625	1.205	0.063	0.588
8.825	1.200	0.090	0.585
8.575	1.199	0.096	0.584
8.775	1.194	0.091	0.582
8.425	1.175	0.097	0.572
6.775	1.170	0.138	0.569
8.675	1.167	0.092	0.568
8.375	1.157	0.099	0.563
8.725	1.154	0.090	0.561
5.475	1.133	0.170	0.550
8.475	1.129	0.091	0.548
2.425	1.124	0.114	0.545
7.525	1.103	0.113	0.534
2.375	1.095	0.118	0.530
8.625	1.074	0.087	0.519
5.225	1.049	0.207	0.506
2.475	1.019	0.098	0.490
8.325	1.018	0.093	0.490
9.675	1.017	0.053	0.489
5.425	1.005	0.157	0.483

*PLS-DA* partial least squares discriminant analysis, *VIP* variable importance in projection

### PLS-DA model permutation

**Fig. 2:**
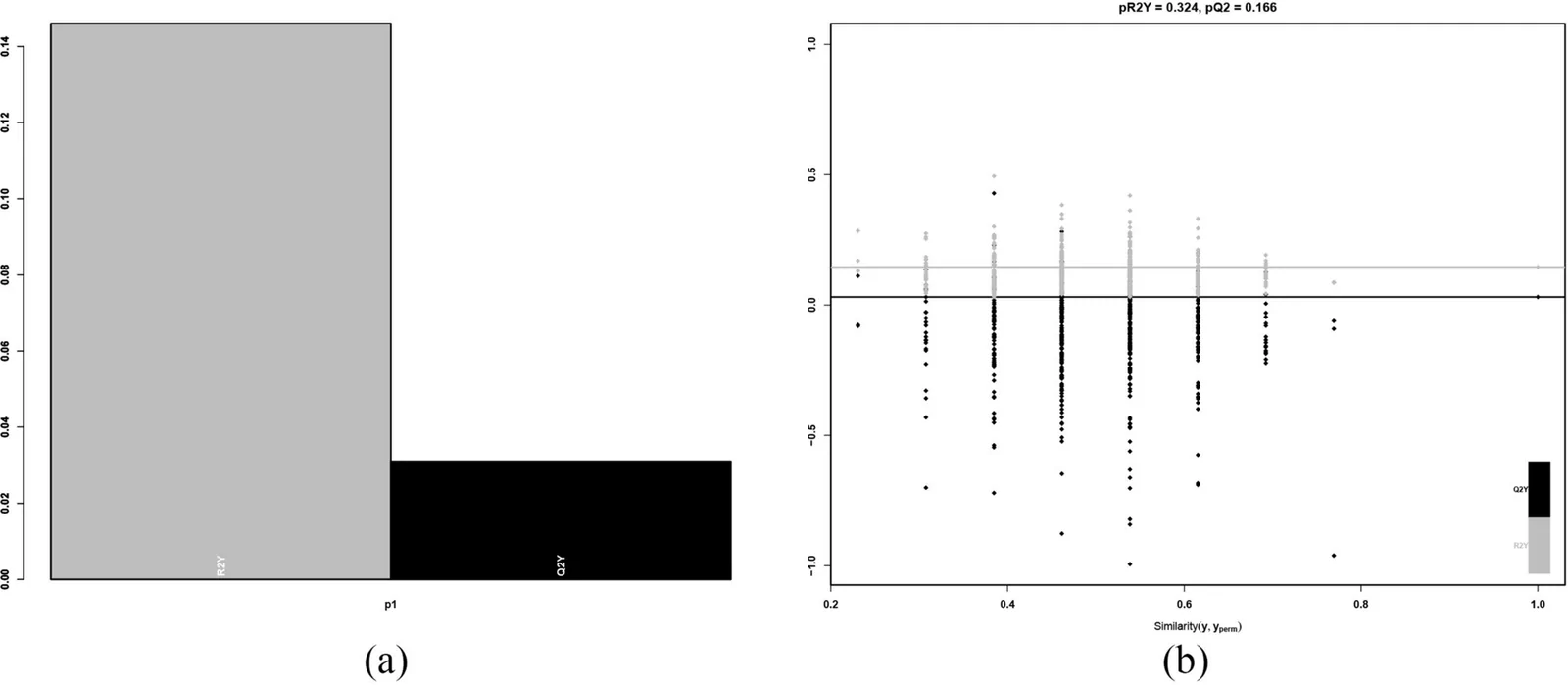
An orthogonal validation and permutation test of the PLS-DA exploratory model (with 500 permutations and 7-fold cross-validation). **a** A summary of the cumulative fit parameters for the one-component cross-validated model, displaying *R*^2^*Y* and *Q*^2^*Y*. **b** A plot of the permutation distribution (with *N* = 500) showing the empirical validation metrics (*pR*^2^*Y* = 0.324, *pQ*^2^ = 0.166)

To test model validity and prevent over-interpretation of classification performance, a 7-fold cross-validation with 500 permutations was evaluated. Permutation testing yielded validation metrics of *pR*^2^*Y* = 0.324 and *pQ*^2^ = 0.166 (Fig. 2). These validation parameters show that, as expected in a pilot cohort of 26 individuals, the global multivariate model on raw spectral bins lacks generalizable out-of-sample classification performance but served as a dimension-reduction and feature-filtering tool. A total of 78 spectral bins exhibited a VIP score >1.0, which were retained for structural annotation and biological pathway mapping.

### Identification of differentially expressed salivary metabolites

Following the preliminary variable selection step, which identified 78 differential spectral bins with VIP scores exceeding 1, metabolite annotation was carried out to assign biological identities to these discriminating features. Candidate metabolites were identified by querying the Biological BMRB and subsequently verified through peak-matching analysis using Chenomx software against a representative ^1^H NMR reference spectrum.

**Fig. 3:**
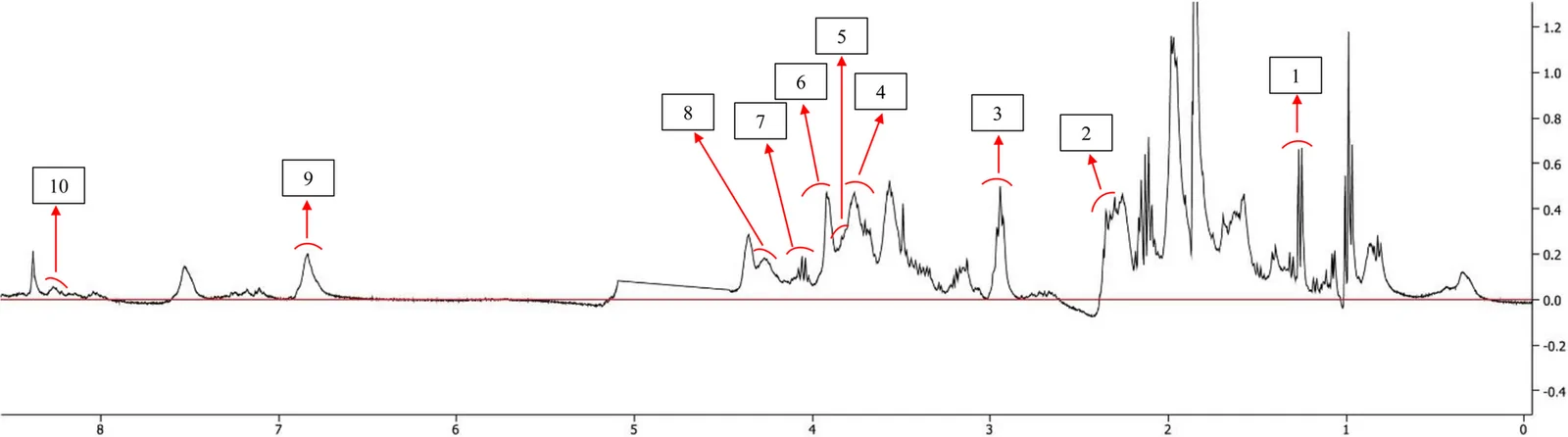
Representative NMR spectrum obtained from one of the samples, shown as a reference image. Based on database searching and verification using Chenomx software, a total of ten peaks were identified. These metabolites are labeled in the figure and include (1) glycolic acid, (2) thymidine, (3) tyramine, (4) deoxyguanosine, (5) cytidine, (6) uridine, (7) NADPH, (8) ADP, (9) serotonin, and (10) nicotinate. The x-axis represents the chemical shift in parts per million (ppm), indicating the specific resonant frequencies of the protons associated with each identified metabolite. The y-axis shows NMR signal intensity in arbitrary units, reflecting relative abundance in the saliva samples. NMR, nuclear magnetic resonance; NADPH, nicotinamide adenine dinucleotide phosphate; ADP, adenosine diphosphate

Through this sequential validation approach, a total of ten metabolites were successfully annotated as differentially expressed when comparing diabetic patients with periodontitis to those without the condition. The metabolites identified were glycolic acid, thymidine, tyramine, deoxyguanosine, cytidine, uridine, NADPH, ADP, serotonin, and nicotinate. A representative ^1^H NMR spectrum highlighting the most characteristic reference peaks for each of these ten annotated metabolites is shown in Fig. 3. As indicated in the Methods section, the peaks emphasized in the figure correspond to the most prominent signals for each compound, confirmed through comprehensive cross-alignment of multiple chemical shifts across the spectral range.

### Enriched pathways

**Fig. 4:**
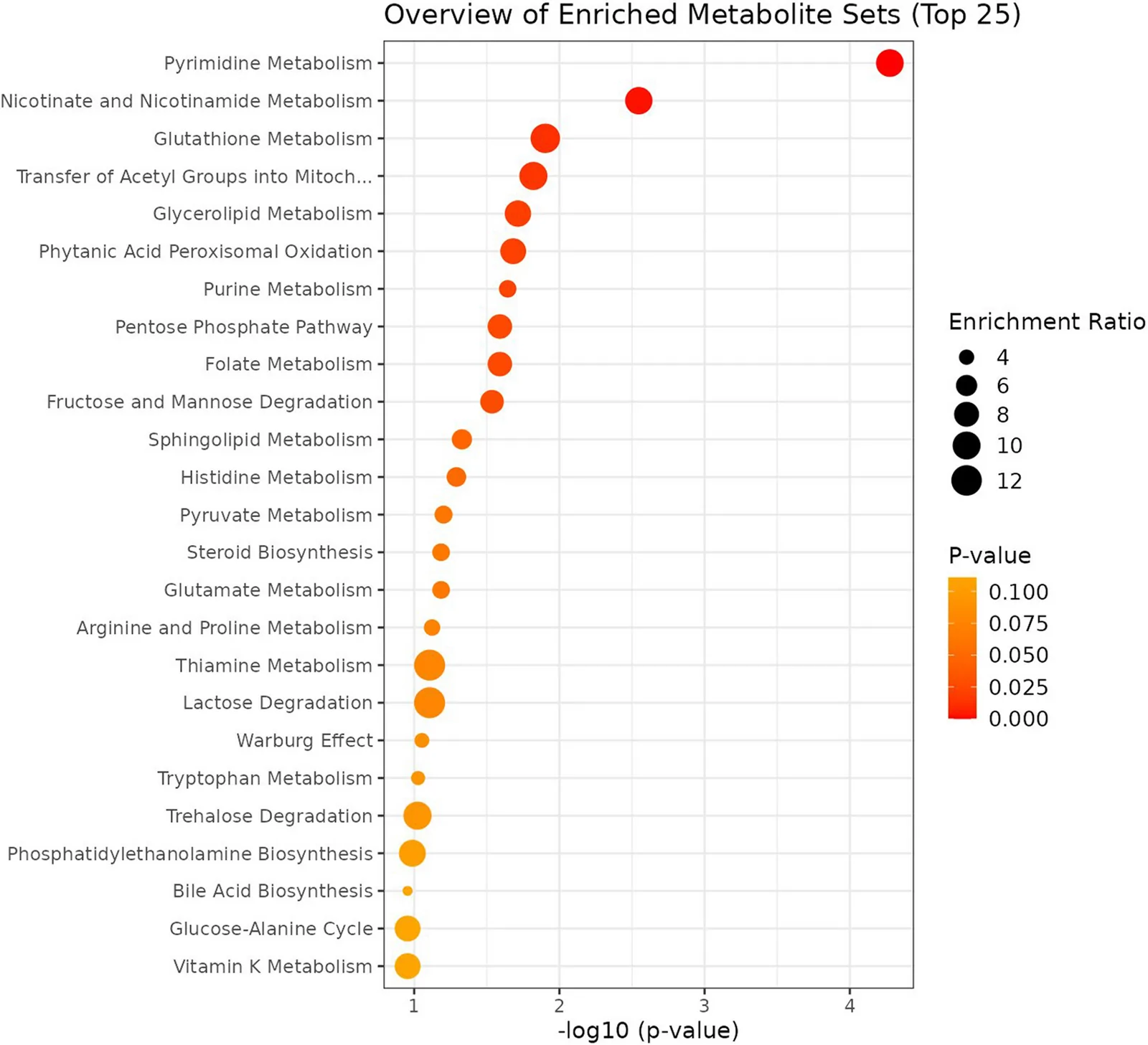
Top 25 enriched metabolic pathways. The bubble plot visualizes pathways by statistical significance (−log10(*p* value) on the x-axis) and enrichment ratio (bubble size)

**Fig. 5:**
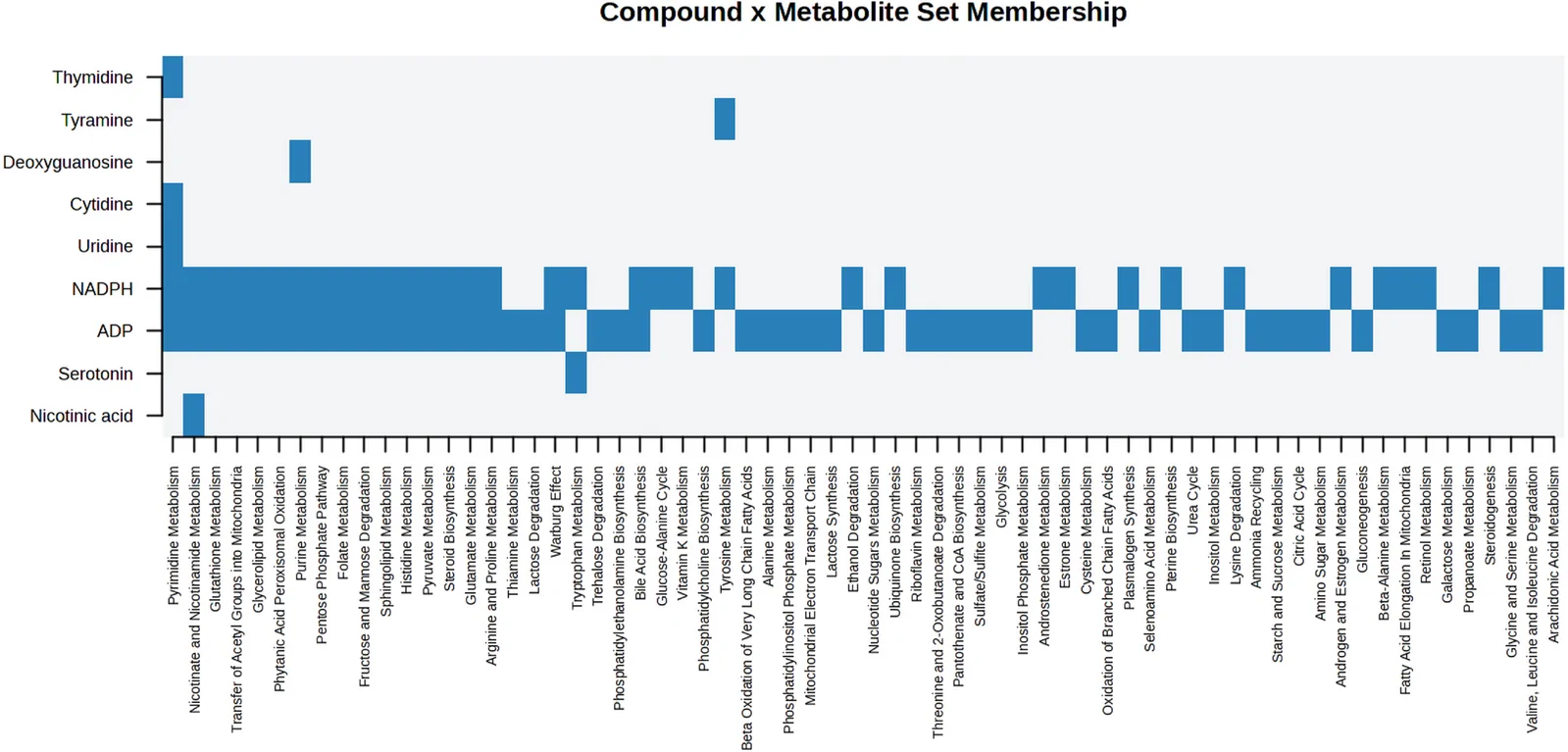
Compound-by-metabolite set membership heatmap. The figure displays the distribution of individual differentially expressed salivary metabolites across the enriched metabolic pathways. Rows are your input compounds, and columns are the top enriched sets ranked left-to-right by *p* value. Blue shading indicates the assignment of a given compound to a particular metabolic set. This visualization reveals that certain metabolites, such as ADP and NADPH, serve as central nodes participating in multiple metabolic routes. In contrast, others, including deoxyguanosine and tyramine, are confined to a single pathway

To achieve deeper insight into the biological relevance and functional meaning of the metabolic differences observed between the two groups, an MSEA was carried out. The top 25 enriched metabolic pathways are displayed in Table 3 and Fig. 4. Only pyrimidine metabolism emerged as a significantly enriched pathway (FDR = 0.005). To further clarify which specific molecules underlie these pathway enrichments, a compound-to-metabolite set membership analysis was conducted to link each altered metabolite to its corresponding biological pathway. Accordingly, five metabolites, including thymidine, cytidine, uridine, NADPH, and ADP, are involved in the pyrimidine metabolism pathway (Fig. 5).

**Table 3:** Top 25 enriched pathways in diabetic patients with periodontitis compared to those without periodontitis

Pathway name	Total	Expected	Hits	Raw *p*	FDR
Pyrimidine metabolism	57	0.512	5	5.30E−05	0.00519
Nicotinate and nicotinamide metabolism	35	0.314	3	0.00284	0.139
Glutathione metabolism	20	0.18	2	0.0125	0.28
Transfer of acetyl groups into mitochondria	22	0.198	2	0.0151	0.28
Glycerolipid metabolism	25	0.225	2	0.0193	0.28
Phytanic acid peroxisomal oxidation	26	0.234	2	0.0208	0.28
Purine metabolism	73	0.656	3	0.0227	0.28
Pentose phosphate pathway	29	0.26	2	0.0257	0.28
Folate metabolism	29	0.26	2	0.0257	0.28
Fructose and mannose degradation	31	0.278	2	0.0291	0.286
Sphingolipid metabolism	40	0.359	2	0.0469	0.417
Histidine metabolism	42	0.377	2	0.0512	0.418
Pyruvate metabolism	47	0.422	2	0.0628	0.426
Steroid biosynthesis	48	0.431	2	0.0653	0.426
Glutamate metabolism	48	0.431	2	0.0653	0.426
Arginine and proline metabolism	52	0.467	2	0.0753	0.426
Thiamine metabolism	9	0.0808	1	0.0783	0.426
Lactose degradation	9	0.0808	1	0.0783	0.426
Warburg effect	57	0.512	2	0.0885	0.436
Tryptophan metabolism	59	0.53	2	0.094	0.436
Trehalose degradation	11	0.0988	1	0.0949	0.436
Phosphatidylethanolamine biosynthesis	12	0.108	1	0.103	0.436
Bile acid biosynthesis	65	0.584	2	0.111	0.436
Glucose-alanine cycle	13	0.117	1	0.111	0.436
Vitamin K metabolism	13	0.117	1	0.111	0.436

*FDR* false discovery rate

## Discussion

Periodontitis is a widespread chronic inflammatory condition affecting the tooth-supporting structures and is well established as the sixth major complication associated with DM. Individuals with diabetes exhibit a considerably higher prevalence and greater severity of periodontal disease, driven by a reciprocal relationship that amplifies systemic inflammation and disrupts immune regulation [31–33].

To better understand the biochemical shifts underlying this heightened vulnerability, the present investigation applied ^1^H NMR-based metabolomics to profile and compare the salivary metabolic composition of diabetic participants with and without periodontitis. By analyzing unstimulated whole saliva, a total of ten differentially expressed metabolites were identified and linked to their corresponding enriched metabolic pathways. These metabolomic signatures offer promising, non-invasive salivary biomarkers that may help identify periodontitis early and stratify risk in diabetic populations. Moreover, these results offer new perspectives on the pathophysiological links between diabetes and periodontal tissue breakdown, with potential implications for designing targeted therapies and individualized patient management.

In this study, multivariate statistical analysis of ^1^H NMR spectral data effectively distinguished the salivary metabolic profiles of diabetic individuals with periodontitis from those without the disease. The results identified ten DEMs responsible for this differentiation: glycolic acid, thymidine, tyramine, deoxyguanosine, cytidine, uridine, NADPH, ADP, serotonin, and nicotinate. To explore the broader biological implications of these metabolic changes, MSEA was conducted on the identified compounds. The pathway enrichment analysis revealed notable disruptions across several biochemical networks, most notably pyrimidine metabolism, which emerged as a key metabolic feature distinguishing periodontal status within this diabetic population.

Several factors limit the generalizability of these findings. First, we recruited participants from a single academic dental center in Hamadan, Iran, using convenience sampling, which may not represent diabetic individuals with periodontitis across different geographic regions, health care systems, or ethnicities. Second, the relatively small sample size (*n* = 13 per group) limits the statistical reliability of the identified metabolic signatures and raises uncertainty regarding their reproducibility in independent datasets. Third, the study population was limited to adults with established type 2 diabetes, and the results may not be directly transferable to patients with type 1 diabetes, prediabetes, or non-diabetic individuals with periodontitis. Additionally, the study did not stratify by periodontitis severity and stage, so the detected metabolic profile may reflect a specific disease burden rather than the full spectrum of clinical presentations. Variations in glycemic control, treatment regimens, dietary patterns, and oral microbiome composition across different populations could also influence the salivary metabolome independently of the periodontitis–diabetes interaction, further complicating direct extrapolation. Therefore, large-scale, multicenter replication in ethnically diverse populations is essential before these metabolic signatures can be considered clinically generalizable or adopted as population-level diagnostic biomarkers.

Wang et al. [34] examined how salivary metabolite profiles evolve across phases of periodontitis. Using gas chromatography-mass spectrometry (GC-MS), the authors analyzed saliva from 116 participants, including 10 healthy controls and 106 patients classified into stages T1 through T4 of periodontitis. Their study revealed progressive, stage-related changes in the concentrations of several discriminatory metabolites, namely glucose, 3-aminobutanoic acid, and methylmalonic acid. The authors also used KEGG pathway analysis to identify associated pathogenic mechanisms. Unlike our study, Wang et al. [34] tracked disease severity across a general periodontitis population using GC-MS; our findings specifically address the metabolic intersection between periodontitis and DM. Moreover, whereas their study highlighted alterations in glucose and amino acid derivatives, our ^1^H NMR-based approach distinctively identified pyrimidine metabolism as a significantly enriched pathway, underscoring the unique salivary metabolic signature that emerges when periodontitis occurs in the context of systemic diabetes.

Tang et al. [35] examined whether metabolic profiles of apical periodontitis lesions differ by lesion size, using this parameter as an indicator of disease severity. The authors used liquid chromatography-mass spectrometry (LC-MS) to examine 12 apical tissue specimens from systemically healthy patients, comparing large lesions (>1000 mm^3^) with smaller ones (<1000 mm^3^). Their analysis uncovered 21 DEMs. Elevated fatty acid derivatives, with enrichment in α-linolenic acid and unsaturated fatty acid biosynthesis, characterized large lesions. In contrast, small lesions showed increased levels of nitrogenous bases and amino acid derivatives linked to purine, glycerophospholipid, and glutathione metabolism.

Chu et al. [36] identified potential metabolic biomarkers and therapeutic targets in gingival tissue from patients with severe periodontitis using LC-MS and GC-MS. Tissue samples from 20 individuals with severe periodontitis and 20 healthy controls were analyzed. Using orthogonal partial least squares discriminant analysis (OPLS-DA) and Student’s *t*-tests, the authors identified 65 DEMs, 40 of which were elevated and 25 reduced, associated with disruptions across 33 metabolic pathways, particularly involving glucose, purine, and amino acid metabolism. From these, 12 candidate biomarkers were identified and further examined for their osteogenic differentiation potential.

Although our study identified alterations in pyrimidine metabolism, Chu et al. [36] and Tang et al. [35] reported changes in purine metabolism. Both pathways are fundamentally linked to the broader nucleic acid metabolic network.

Jiang et al. [37] analyzed the subgingival microbial and metabolic profiles of patients with type 2 DM, with and without periodontitis, and examined their associations with T lymphocyte-mediated immunity. Using 16S rRNA gene sequencing, UHPLC-MS-based metabolomics, and flow cytometry, Jiang et al. [37] compared diabetic and non-diabetic groups. Their findings revealed that periodontitis-related shifts in the microbiome and metabolome were modified in the context of type 2 diabetes. These changes were accompanied by alterations in the Treg/Th17 balance and immune checkpoint markers, suggesting a disrupted interplay among bacterial communities, metabolic pathways, glycemic status, and immune regulation.

Pyrimidine metabolism appeared to be linked to periodontitis, as genes associated with pyrimidine degradation were enriched in diseased subgingival plaque. In contrast, genes involved in pyrimidine synthesis showed lower relative abundance than in healthy controls [38].

Cai et al. [39] explored whether a bidirectional causal relationship exists between specific blood metabolites and chronic periodontitis using a two-sample Mendelian randomization (MR) framework. Their analysis used genetic instruments for 1400 blood metabolites derived from 8299 participants, along with chronic periodontitis data from the FinnGen cohort (4784 cases and 272,252 controls). The inverse-variance weighted (IVW) MR method was the primary analytical approach, complemented by sensitivity analyses and metabolic pathway enrichment to identify relevant metabolite–disease connections. Their findings highlighted five blood metabolites with the strongest associations: butyrylglycine, orotidine, 2′-deoxyuridine, N-stearoyl-sphinganine, and maltotriose. Among these, 2′-deoxyuridine, a product of pyrimidine metabolism, is primarily involved in DNA synthesis and repair. Notably, a multifluid metabolomic investigation of periodontal disease linked 2′-deoxyuridine levels to periodontitis, suggesting that changes in plasma concentrations of this metabolite may correlate with disease initiation and progression [40]. Given its established role in cell proliferation and nucleic acid metabolism, Cai et al. [39] proposed that 2′-deoxyuridine could contribute to the development of chronic periodontitis by modulating cell proliferation and inflammatory pathways.

While the current investigation provides valuable perspectives on the salivary metabolome in diabetic patients with periodontitis, several limitations should be taken into account. The data were obtained using a 400-MHz spectrometer. Although this field strength was adequate for the present study, higher field strengths (for example, 600 MHz) provide better spectral dispersion and sensitivity, helping resolve overlapping resonances in complex biofluids like saliva. It is therefore possible that some of the signals have been only partly overlapped or have been assigned with less confidence. Metabolite identification and quantification could be improved in future studies that make use of higher-field instruments. The limited sample size reduces statistical power and restricts the generalizability of the results to broader populations. The PLS-DA model had low cross-validated predictive capability, and ROC analysis was not performed on independently collected data. The global model has limited generalizable predictive power (*Q*^2^ = 0.031; *pQ*^2^ = 0.166), and an AUC of 0.905 indicates in-sample group separation, not prospective classification performance. As a result, the model is based on separations observed in this sample and may be overfit. Therefore, the 78 VIP variables, together with the ten differential metabolites, should be considered potential markers until they are validated in an independent cohort. In addition, the cross-sectional design does not allow causal interpretation of the relationship between the observed metabolic changes and the initiation or progression of periodontitis in the context of diabetes mellitus. Longitudinal studies are needed to track metabolic variations over time. In addition, while the current study design allows an effective comparison between diabetic patients with periodontitis and those without, it does not include non-diabetic healthy controls or non-diabetic individuals with periodontitis. As a result, it is unclear whether the ten metabolites identified are specific markers of the diabetes–periodontitis interaction or general markers of periodontitis that might be found in anyone. This limitation must be considered when interpreting the results and when planning future studies that include additional control groups to isolate the metabolic signature of diabetic periodontitis. Furthermore, although ^1^H NMR spectroscopy offers high reproducibility, quantitative accuracy, and non-destructive analysis, its lower sensitivity than mass spectrometry-based techniques may have limited detection of trace-level metabolites. Several sources of potential bias also merit consideration. Participants were recruited through convenience sampling from a single academic institution, which may affect representativeness and introduce selection bias; individuals consenting to participate might differ systematically from non-participants in terms of health-related behaviors or disease severity. Although both the examiner and the analyst were blinded to group allocation, self-reported medical history and lifestyle information were not independently confirmed, potentially introducing information bias. No multivariable adjustments were made for potential confounders—such as age, sex, smoking, HbA1c, BMI, diabetes duration, or medication use, owing to the exploratory design and small cohort size. Consequently, residual confounding related to glycemic control, concomitant medications, or systemic inflammation cannot be excluded. The direction and magnitude of any such bias remain uncertain; uncontrolled confounding may either inflate or attenuate the true metabolic differences between groups. The small sample also yields wide confidence intervals around effect estimates and limited predictive accuracy, as reflected in the low *Q*^2^ value. Moreover, the observed AUC of 0.905 may be overly optimistic due to potential overfitting and will require external validation. Lastly, the applicability of these results to non-diabetic populations, other ethnic groups, or patients with varying stages of periodontitis or glycemic control remains unclear. To address these limitations, future research should include large-scale, multicenter cohort studies using a multi-omics approach to validate these findings and establish the clinical relevance of the identified metabolic signatures.

## Conclusion

^1^H NMR analysis of saliva metabolites distinguished diabetic patients with periodontitis from those without. Although the PLS-DA model separated the two groups in this small exploratory sample, its low cross-validated predictive power (*Q*^2^ = 0.031) suggests it serves mainly as a feature-selection tool rather than a clinical diagnostic classifier. This approach helped identify downstream metabolites and enrichment of the pyrimidine metabolism pathway, highlighting candidate biomarkers likely indicative of increased cellular turnover, tissue destruction, and inflammation in diabetes-associated periodontitis. Because the comparison was limited to diabetic patients, the identified metabolites may mark periodontitis in this group. Still, their broader specificity must be determined in future studies that include healthy controls and periodontitis-only groups. While these findings improve our understanding of the connections between oral and systemic diseases, candidate biomarkers must be independently validated across multiple centers before clinical use, opening the way for further longitudinal studies designed to enable early, non-invasive diagnosis and targeted therapies.

## Supplementary Information

Additional file 1: Supplementary Data 1. 1H NMR spectra of saliva samples using ProMetab software. The figure illustrates the sequential data preprocessing steps applied to the salivary metabolomic dataset of diabetic patients with and without periodontitis before multivariate statistical analysis. The panels display: (a) untransformed normalized binned NMR spectra; (b) peak-aligned untransformed normalized raw NMR spectra; (c) shift-aligned normalized NMR spectra, correcting for minor variations in chemical shifts; (d) raw normalized NMR spectra; and (e) raw unnormalized NMR spectra following baseline offset correction. Data processing was performed to minimize non-biological variations and prepare the spectra (0.2–10 ppm) for subsequent partial least squares discriminant analysis. PLS-DA, partial least squares discriminant analysis; NMR, nuclear magnetic resonance. Supplementary Data 2. PLS-DA model quality parameters derived from 1H NMR data. The bar chart shows cumulative R2X (0.449), R2Y (0.146), and Q2 (0.031), representing the model’s ability to distinguish diabetic patients with periodontitis from those without. PLS-DA, partial least squares discriminant analysis; 1H NMR, proton nuclear magnetic resonance.

## Data Availability

Data available on request due to privacy/ethical restrictions.
