# Supplementary material for: Salivary 1H NMR metabolic profiling of diabetic patients with and without periodontitis: an exploratory pilot study
Source: Genomics Inform. 2026 Oct 26;24:23. doi: 10.1186/s44342-026-00085-4 (PMC13700260; doi:10.1186/s44342-026-00085-4)
Supplement: Supplementary file 1 [file 44342_2026_85_MOESM1_ESM.docx]

**Supplementary Data 1. ^1^H NMR spectra of saliva samples using ProMetab software.** The figure illustrates the sequential data preprocessing steps applied to the salivary metabolomic dataset of diabetic patients with and without periodontitis before multivariate statistical analysis. The panels display: **(a)** Untransformed normalized binned NMR spectra; **(b)** Peak-aligned untransformed normalized raw NMR spectra; **(c)** Shift-aligned normalized NMR spectra, correcting for minor variations in chemical shifts; **(d)** Raw normalized NMR spectra; and **(e)** Raw unnormalized NMR spectra following baseline offset correction. Data processing was performed to minimize non-biological variations and prepare the spectra (0.2–10 ppm) for subsequent partial least squares-discriminant analysis. PLS-DA, partial least squares discriminant analysis; NMR, nuclear magnetic resonance.

**Supplementary Data 2.** PLS‑DA model quality parameters derived from ¹H NMR data. The bar chart shows cumulative R²X (0.449), R²Y (0.146), and Q² (0.031), representing the model's ability to distinguish diabetic patients with periodontitis from those without. PLS-DA, partial least squares discriminant analysis; ¹H NMR, proton nuclear magnetic resonance.
